# Molecular Subgroups of Diffuse Large B Cell Lymphoma: Biology and Implications for Clinical Practice

**DOI:** 10.1007/s11912-021-01155-2

**Published:** 2022-01-20

**Authors:** Theresa Weber, Roland Schmitz

**Affiliations:** 1 Department of Hematology, Oncology and Immunology, University Hospital Giessen and Marburg, Philipps University Marburg, Baldingerstrasse, 35033 Marburg, Germany; 2 Institute of Pathology, Justus Liebig University Giessen, Langhansstrasse 10, 35392 Giessen, Germany

**Keywords:** Lymphoma, DLBCL, Gene expression profiling, Cell-of-origin classification, ABC, GCB, Molecular classification, High-throughput sequencing, High-grade lymphoma, Double-hit lymphoma, Genetic subclassification, ctDNA, Precision medicine

## Abstract

**Purpose of Review:**

Genomic analyses have immensely advanced our conception of the heterogeneity of diffuse large B cell lymphoma (DLBCL), resulting in subgroups with distinct molecular profiles. In this review, we summarize our current knowledge of the biology of DLBCL complexity and discuss the potential implications for precision medicine.

**Recent Findings:**

During the last two decades, gene expression profiling, copy number analysis, and high throughput sequencing enabled the identification of molecular subclasses of DLBCL that are biologically and clinically meaningful. The resulting classifications provided novel prospects of diagnosis, prognostication, and therapeutic strategies for this aggressive disease.

**Summary:**

The molecular characterization of DLBCL offers unprecedented insights into the biology of these lymphomas that can guide precision medicine. The knowledge of the molecular setup of an individual DLBCL patients enables prognostication of patients and will be useful to stratify patients in clinical trials. Future direction should focus to implement the molecular classifications of DLBCL in the clinical practice to evaluate their significance and scope using real-world data.

## Introduction

Diffuse large B cell lymphoma (DLBCL) is the most common type of B cell non-Hodgkin lymphoma [1]. This aggressive lymphoma has a high clinical heterogeneity manifesting in diverse responses to rituximab plus chemotherapy (R-CHOP). Early prognostic stratification of DLBCL was developed almost 30 years ago by Shipp and colleagues by combining several clinical parameters to divide patients into risk groups [2]. The resulting International Prognostic Index (IPI) has become the most commonly used prognostic score for aggressive lymphomas [2–4]. The first classification of DLBCL based on molecular traits dates back 20 years, when Alizadeh et al. analyzed 96 samples of normal and malignant lymphocytes by gene expression profiling (GEP) using DNA microarrays. This hallmark study identified two subtypes of DLBCL that were distinguished by the expression of genes typical for normal germinal center B cells or activated blood B cells prompting the cell-of-origin classification into ABC and GCB DLBCL [5]. Notably, 10–15% of DLBCL cannot be categorized into one of the two groups and are therefore termed unclassified DLBCL. Importantly, this gene expression-based concept was able to define prognostic categories, which allowed the identification of high-risk disease independently from the IPI. In the R-CHOP era, several GEP studies confirmed the prognostic impact and showed that ABC DLBCL encompass the highest risk for relapse and an inferior outcome [6, 7]. Consequentially, the 2016 revision of the World Health Organization classification of lymphoid neoplasms requires the identification of molecular subgroups of DLBCL [1].

In the recent past our knowledge of the biology and heterogeneity of DLBCL advanced enormously by the application of genomic technologies for high throughput analysis. Several mutations and other structural changes in the cancer genome have been characterized that can serve as diagnostic and prognostic markers. In this review, we will summarize our knowledge of the biology underlying the heterogeneity of DLBCL and illustrate how the classification of DLBCL subgroups using comprehensive molecular characterization can enhance diagnosis and prognostication of this aggressive lymphoma.

## Biology of the Cell-of-Origin Classification

Gene expression profiling using DNA microarrays represents one of the earliest technologies for genomic high throughput analysis. About 20 years ago, this technique was used to characterize the abundance of transcripts of several thousand genes in subsets of normal B cells and DLBCL samples [5]. This comparison identified a group of DLBCL that express genes characteristic for normal germinal center (GC) B cells and a group of DLBCL that resembled in their gene expression program blood B cells activated by engagement of B cell receptor and stimulated with varying combination of CD40 ligand and IL4. Thus, due to the similarities of the gene expression profiles of these tumor to normal B cells, the two subgroups have been termed germinal center B cell like (GCB) and activated B cell like (ABC) DLBCL [5].

The gene expression patterns observed in a proportion of samples do not allow a classification of these lymphomas into the ABC or GCB subgroup. These cases are consequently designated as unclassified DLBCL, which is why unclassified DLBCL are often mistaken for a distinct, third group defined by gene expression. However, these cases represent an aggregation of cases that do not fit the model distinguishing ABC and GCB DLBCL [8]. Notably, recent analysis indicated that a substantial number of unclassified DLBCL bear a distinctive signature of mutations and structural gene aberration indicating the existence of a molecular subgroup within unclassified DLBCL (see below).

Gene expression analysis offers a view into tumor phenotypes. These phenotypes are dictated by the accumulation of genetic and epigenetic lesions in the course of malignant transformation of a normal B cell into an aggressive lymphoma. Studies investigating the tumor genome of DLBCL validated this concept identifying several genetic lesions that are restricted to either subtype [9–15, 16••]. Frequent genetic alterations that are almost exclusively found in GCB DLBCL are chromosomal translocations of the *BCL2* locus, oncogenic mutations in the *EZH2* gene, amplification of the *REL* locus and mutations or deletions in *PTEN* [9–11, 17]. While loss of function of *PTEN* activates the PI3 kinase (PI3K) pathway, increased expression of REL due to amplification likely contributes to the GC B cell phenotype of GCB DLBCL as REL was found to be required for GC maintenance [18]. Likewise, the EZH2 protein, a subunit of the polycomb repressive complex, is involved in germinal center B cell biology. This gene that is required for GC formation contributes to the phenotype of GC B cells by repressing proliferation checkpoint genes and establishing bivalent chromatin domains thereby enabling a lineage specific (i.e., GC B cell) gene expression program [19, 20]. The oncogenic translocation *t*(14;18) leads to the transcriptional deregulation of *BCL2* gene, positioning the coding exons next to regulatory elements of the immunoglobulin heavy chain locus [21]. Interestingly, although *BCL2* translocations are almost exclusively found in GCB DLBCL, ABC tumors express high level of *BCL2* transcripts, indicating other pathogenetic mechanisms e.g. amplification of the *BCL2* locus observed in approximately 30% of ABC DLBCL [22, 23]. Other genetic events characteristic for ABC DLBCL involve components of the B cell receptor (*CD79A* and *CD79B*) and regulators of the NF-κB pathway (*MYD88*) [12, 13]. Recent studies revealed that both mutations collaborate in forming a multiprotein complex that promotes the constitutive activation of the NF-κB transcription factor complex through chronic B cell receptor (BCR) signaling [24, 25]. The central relevance of the BCR signaling in the biology of ABC DLBCL, explains the phenotype of activated B cells and further validates the biologic concept of the cell-of-origin classification.

## Clinical Significance of the Cell-of-Origin Classification

The initial classification of DLBCL based on gene expression profiling revealed a significant survival difference between the subgroups, indicating an inferior outcome for patients with ABC DLBCL versus GCB DLBCL. Unclassified cases showed an intermediate survival rate [5, 8, 23]. These results prompted several investigations as to how the cell-of-origin classification could be implemented in clinical practice using techniques widely available in routine diagnostics. Hence, alternative methods using immunohistochemical markers as surrogates for the expression programs distinguishing DLBCL subgroups have been developed [26–29]. The method that became the most widely accepted was described by Hans and colleagues, classifying DLBCL by immunohistochemistry staining of CD10, BCL6, and MUM1 into a GCB and a non-GCB subgroup. However, besides variable concordance to gene expression profiling for the tested algorithms, these methods showed highly variable results for interlaboratory comparisons [26, 28, 30–32]. To overcome these issues, Scott and colleagues presented a novel, commercially available assay for subclassification based on gene expression of 20 genes that is applicable to formalin-fixed paraffin-embedded tissue [31]. Tests of the Lymph2Cx assay showed high concordance when compared to gene expression profiling (GEP) using DNA microarray analysis on RNA derived from frozen DLBCL biopsies.

Given its major biological and clinical significance, several clinical trials integrated the cell-of-origin classification [33–37]. While some studies confirmed the survival difference observed for patients with ABC, GCB, or UC DLBCL [33, 34], other analyses achieved varying results. In 2013, a UK trial observed no survival differences of patients treated with R-CHOP using the immunohistochemistry based classifier [35]. However, a subsequent reanalysis with microarray-based subclassification revealed that the ABC subtype of DLBCL is independently associated with inferior survival than GCB DLBCL [36]. In 2017, the Lymph2Cx assay was used to classify more than 400 DLBCL patients from two German clinical trials treated chemotherapy and/or immunochemotherapy [37]. While the cell-of-origin classification disclosed prognostic subgroups in patients treated with CHOP, no such survival difference was observed for R-CHOP–treated patients. The stark contrast of these results and the conclusions from population-based studies might be explained by different patient cohorts analyzed [38]. Although rigorously supervised, patient enrolled in randomized controlled trials might not always fully represent the overall patient population and have a lower risk profile than real-world populations [39].

## Risk Stratification Using Structural Genetic Lesions: High Grade B Cell Lymphoma

The majority of recurrent genetic alterations in DLBCL differs significantly in their prevalence in ABC and GCB DLBCL, suggesting that the biological and clinical phenotype of DLBCL can be dictated by genetic variants. Chromosomal translocations juxtaposing the oncogenes *MYC, BCL2*, or *BCL6* and one of the immunoglobulin loci are hallmarks in the genesis of lymphomas [21]. Thus, several studies investigated whether these structural aberrations could serve as biomarkers for DLBCL prognostication. *MYC* rearrangements occur in approximately 10% in DLBCL [40•]. In contrast to Burkitt lymphoma (BL), where *MYC* is almost exclusively fused to an immunoglobulin locus, up to 50% of *MYC* translocations in DLBCL involve non-immunoglobulin genes, including among others *BCL6*, *PAX5*, and *IKZF1* [41, 42]. Several studies indicated that a translocation of *MYC* is a strong adverse prognostic factor demonstrating an inferior overall (OS) and progression free survival (PFS) compared to patients without *MYC* rearrangement [43–45]. A recent study resolved the role of *MYC* rearrangements as a biomarker for predicting outcome of DLBCL. The negative prognostic impact of *MYC* rearrangements in DLBCL is largely observed in patients with translocations of *MYC* in combination with *BCL2* and/or *BCL6* in which *MYC* is translocated to an IG partner [40•]. Co-occurrence of rearrangements of *MYC* and *BCL2* and/or *BCL6* translocations was reported in 2–8% of all DLBCL including patients from clinical trials and a population-based registries [40•, 46]. These so-called “double-hit” or “triple-hit” (DH/TH) lymphomas have been included in the 2016 updated WHO classification in the new category of high-grade B cell lymphoma (HGBL) with rearrangements of *MYC* and *BCL2* and/or *BCL6* [1]. HGBL-DH/TH lymphoma primarily have a GCB DLBCL phenotype, perhaps not surprising given that *BCL2* translocations are almost exclusively found in GCB DLBCL [46].

Two recent studies extended the definition of HGBL using gene expression profiling to identify DLBCL with inferior prognosis beyond the survival distinctions associated with the cell-of-origin classification [47••, 48••]. The study by Sha et al. used a gene expression signature composed of genes highly expressed in BL compared to DLBCL to define cases of molecular HGBL. Whereas, Ennishi et al. focused on GCB DLBCL to develop a signature derived from genes differentially expressed between *MYC*/*BCL2* double-hit and non-double-hit GCB-DLBCLs. Notably, both signatures were capable of identifying most double-hit lymphomas. However, about half of the DLBCL from the identified the high risk group did not harbor gene rearrangements of *MYC* and *BCL2*, suggesting the existence of alternative genetic or epigenetic alterations causing a high-grade lymphoma phenotype.

A number of studies have indicated that patients with DLBCL that lack *MYC* and *BCL2* rearrangements, but have high expression of both MYC and BCL2 proteins have an inferior outcome [37, 45, 49]. These so called double expressor (DE) DLBCL are primarily found to be of ABC DLBCL phenotype, suggesting alternative mechanisms responsible for upregulation of BCL2 and MYC protein expression (e.g., chromosomal amplification of the gene *BCL2* locus or the activation of the NF-κB pathway) [24]. Compared to HGBL-DH DLBCL, DE-DLBCL have a better overall survival with R-CHOP therapy. Variable clinical outcomes have been reported for DE-DLBCL when compared to non-DE DLBCL. While in elderly patients DE-DLBCL status versus non-DE DLBCL was associated with inferior prognosis [37], other studies did not confirm a prognostic significance of this classification [50], including studies investigating a survival difference in young patients [51], and in patients with stage I/II DLBCL [52].

## Genetic Subclasses of DLBCL

The molecular classification of DLBCL based on phenotypic features such as characteristic gene expression signatures or specific chromosomal translocations deconstructed some heterogeneity of clinical outcome. However, while some of the designated high-risk patients are cured, other patients with a predicted positive prognosis succumb to the disease, indicating that these models do not fully account for the heterogeneous responses to chemotherapy. These considerations provided the rationale for multiplatform genomic analyses integrating DNA copy number alterations, chromosomal translocations, recurrent mutations and gene expression profiling that resulted in a novel genetic subclassification of this disease. Remarkably, two independent studies presented a very similar genetic taxonomy although largely distinct mathematic algorithms were used to group DLBCL tumors into genetic subtypes [16••, 53••].

To distinguish molecular subtypes Chapuy and colleagues analyzed 304 DLBCL biopsies to identify candidate cancer driver genes. To this end, the study combined algorithms that classify mutations occurring more often than expected by chance (MutSigCV), mathematical models that identify clustering of missense mutations in 3-dimensional protein structures (CLUMPS), and a model to identify significant copy number variants and structural aberrations [54–56]. Using clustering of the 158 identified genetic driver alterations resulted in five clusters, termed C1–C5, each with a discrete genetic signature, as well as a cluster without any detectable alterations [53••]. In contrast, the parallel genomic DLBCL analysis classified 574 tumors into genetic subtypes using the GenClass algorithm that starts with an initial set of genetic aberrations and then iteratively examines all possibly re-assortments of cases into classes to optimize for genetic distinctiveness [16••]. The genetic alterations that define the seed classes of this taxonomy were significantly more prevalent in either ABC, GCB, or unclassified DLBCL. This approach distinguished four genetic subtypes, termed MCD, BN2, N1, and EZB. This terminology refers to the seed alterations that define the genetic subgroups: *MYD88*^L265P^ and *CD79B* mutations in MCD, *BCL6* translocations and *NOTCH2* mutations in BN2, *NOTCH1* mutations in N1, and *EZH2* mutations and *BCL2* translocations in EZB. A subsequent study extending the GenClass algorithm on genomic data from more than 1200 DLBCL added two novel genetic subclasses [57••]: A53 defined by cases with *TP53* inactivation associated with aneuploidy and the ST2 subgroup for tumors characterized by the seed class harboring mutations in *SGK1* and *TET2*. The two different algorithmic models used (i.e. consensus clustering and GenClass) resulted in genetic subgroups that had similar genetic signatures (Table 1). Remarkably, both classifications revealed that DLBCL patients subdivided into molecular groups had significantly different clinical outcomes, highlighting that this novel molecular taxonomy is biologically and clinically meaningful [16••, 53••].

**Table 1:** Biological and clinical feature of genetic subclasses of DLBCL

Genetic subgroup (GenClass algorithm)	Corresponding subgroup (consensus clustering)	Prevalence	Cell of origin subtype	Distinctive genetic lesions	Affected oncogenic pathways	5-year OS	Genetically related lymphomas
MCD	C5	14%	ABC99% UC1% GCB0%	MYD88^L265P^, CD79B, CD79A CDKN2A CD274, PDCD1LG2, CD58, HLA-B, -A, -C BCL2 PRDM1, SPIB, IRF4	BCR dependent NF-κB signaling Cell cycle control Immune evasion Apoptosis B cell differentiation	40%	primary extranodal lymphomas
N1	–	3%	ABC94% UC6% GCB0%	NOTCH1 ID3, BCOR	NOTCH1 signaling B cell differentiation	27%	NOTCH1-mutant CLL
A53	C2	7%	ABC76% UC5% GCB19%	TP53, TP53BP, TP73, ING1 B2M	P53 pathway/cell cycle regulation DNA damage response Immune evasion	63%	-
BN2	C1	16%	ABC40% UC42% GCB18%	NOTCH2, SPEN PRKCB, BCL10, TNFAIP3, TNIP1 CD70 CCND3	NOTCH2 signaling BCR-dependent NF-κB pathway Immune evasion Cell cycle control	67%	MZL
ST2	C4	5%	ABC22% UC22% GCB56%	SOCS1, DUSP2, STAT3 SGK1, P2RY8	JAK/STAT signaling PI3K/AKT signaling	84%	NLPHL
EZB	C3	13%	ABC5% UC9% GCB86%	EZH2, KMT2D, CREBBP, EP300, ARID1A PTEN, S1PR2, GNA13 BCL2, FAS IRF8, MEF2B, REL	Epigenetic deregulating PI3K signaling Apoptosis GC B-cell differentiation	68%	FL

Prevalence was calculated using the cohort studied in [16••]. Note that 6% genetically composite DLBCL and 36% other (non-subtyped) DLBCL are not shown [57••]. Overall survival based on all DLBCL. *CLL* chronic lymphocytic leukemia, *MZL* marginal zone lymphoma, *NLPHL* nodular lymphocyte predominant Hodgkin lymphoma, *FL* follicular lymphoma

The MCD genetic subtype (and its corresponding cluster C5) is primarily composed of ABC DLBCL. Within ABC DLBCL, MCD subtypes had significantly inferior survival as compared with other genetic subgroups or with patients with ABC tumors that were not genetically classified due to lack of sufficient distinguishing genetic events. Genetic hallmarks of the MCD subtype are *MYD88*^*L265P*^ and *CD79B* mutations, which cooperatively activate NF-κB signaling [25]. Tumors of this subtype frequently delete the *CDKN2A* locus, which encodes the cell cycle inhibitor p16, which likely accelerates proliferation in MCD tumors [56]. Frequent chromosomal amplification of the *BCL2* locus induces upregulation of this anti-apoptotic protein contributing to the sustained viability. About 7 out of 10 MCD DLBCL harbor genetic aberrations affecting one or more genes encoding immune regulators, which enables immune evasion. These genetic lesion include inactivation of MHC class I genes, inhibiting MHC class antigen presentation, gene fusions that elevate expression of *CD274* and *PDCD1LG2* (encoding PD-L1 and PD-L2) thereby decreasing T cell activation, and mutations and deletions of *CD58*, causing diminished NK cell activation [58]. A number of genetic alterations that define the MCD genetic subtype are also recurrently mutated in primary extranodal lymphomas originating in the CNS, skin, testis, breast and intravascular space [57••]. Interestingly, many MCD DLBCL (as well as C5 cases) are associated with extranodal involvement, affecting particularly the CNS and testis [16••, 53••, 57••]. The CNS and the testis are classical sites of immunologic privilege, raising the possibility that the tropism of MCD tumors to these sites is one mechanism of escape from immunologic surveillance that is amplified by the acquisition of genetic lesions affecting immune recognition (Table 1).

The A53 subtype (and the corresponding cluster C2) is characterized by mutations and deletions of *TP53*. A53 tumors also frequently inactivate *TP53BP1*, which encodes a sensor of DNA damage that synergizes with p53 to suppress genomic instability [59]. Interestingly, lymphomas of this genetic subtype harbor gains and losses of multiple chromosomal regions, including copy number changes of the whole arm of various chromosomes. Focal deletions target the tumor suppressors *TP73*, a p53 family member, and *ING1*, a component of the p53 signaling pathway [60]. The A53 subtype is enriched for homozygous deletions and truncating mutations targeting the MHC class I subunit, *B2M*, providing a mechanism of escape from immune surveillance [58]. The A53 genetic subtype is dominated by ABC DLBCL. Compared with tumors of this molecular subclass, A53 had an inferior survival than the BN2 group, but no significant survival difference to MCD or N1 was observed (Table 1).

The least prevalent subtype, N1, which does not have an equivalent in the molecular classification using consensus clustering, is characterized by gain-of-function NOTCH1 mutations that remove all or part of the C-terminal PEST domain. These NOTCH1 variants appear to have similar function to those observed in chronic lymphocytic leukemia and mantle cell lymphoma, but are distinct from the membrane-proximal NOTCH1 mutations in T-cell acute lymphoblastic leukemia [61]. This subtype is almost exclusively comprised of ABC DLBCL. Within this subgroup patients with N1 have significantly inferior survival as compared with patients with BN2 or ABC tumors that were not genetically classified.

The BN2 subtype (and the C1 cluster) is characterized by *BCL6* translocations, activating mutations of *NOTCH2*, and by inactivating of *SPEN*, a nuclear repressor that antagonizes NOTCH-dependent gene activation. Further prominent genetic features of this subgroup were mutations in genes encoding regulators of B cell receptor (BCR) signaling such as *PRKCB* and *BCL10*, as well as inactivating lesions in *TNFAIP3* and *TNIP1*, encoding inhibitors of the NF-κB pathway [62]. BN2 has a relatively equal contributions from unclassified and ABC DLBCL, but also, to lesser extent, from GCB DLBCL. Remarkably, BN2 is the genetic subtype that represents most of the DLBCL that were unclassified by gene expression profiling. Comparing all cases, survival of BN2 was favorable in relation to MCD and N1, but not significantly different to any other genetic subtypes (Table 1). However, when comparing within ABC, BN2 was favorable compared all non-BN2 [57••]. Several mutations characteristic of BN2 subtype occur frequently in marginal zone lymphomas. This includes mutations in *NOTCH2* and *SPEN*, which are involved in differentiation of follicular B cells into marginal zone B cells, as well as aberrations in *BCL10*, *TNFAIP3*, and *TNIP1* [63].

The ST2 genetic subtype is characterized by mutations in *SGK1* and *TET2*. TET2 is an epigenetic regulatory enzyme that catalyzes the hydroxylation of methylated cytosine residues in DNA. ST2 tumors harbor inactivating mutations in this gene in keeping with its ability to promote germinal centers when disabled in mice [64]. Mutations in SGK1, a kinase in the AKT family that is activated by many cellular responses, likely modulates PI3K signaling in these tumors. Although *SGK1* is a target for SHM, preferential acquisition of loss-of-function mutation suggests that it may function as a tumor suppressor in ST2. JAK/STAT signaling is likely the target of several ST2-defining lesions, including inactivation of *SOCS1*, a negative regulator of JAK signaling as well as activating mutations in *STAT3* and inactivation of *DUSP2*, a phosphatase that controls the activity of STAT3 [65]. The ST2 genetic subtype is predominantly composed of GCB DLBCL. Compared to GCB DLBCL the survival of this subtype is favorable to the EZB group (Table 1).

The genetic subgroup of EZB DLBCL, as well as the corresponding cluster C3, is almost exclusively composed of GCB DLBCL. Consequentially, the defining features of the EZB genetic subgroup are enriched in GCB DLBC, including translocations of *BCL2*, amplification of the *REL* locus, inactivation of *PTEN*, and activating mutations of the histone methyltransferase *EZH2*. Remarkably, mutations of several other epigenetic regulators such as *KMT2D*, *CREBBP*, *EP300*, and *ARID1A* are significant features of the genetic signature of this subtype [16••, 53••, 57••]. The perturbation of the epigenetic homeostasis in EZB tumors likely cooperates with mutations in genes encoding factors controlling germinal center B cell development such as IRF8, MEF2B, S1PR2, and GNA13, thus enabling a GC B cell transcriptional profile [66–68]. Among GCB cases, EZB had an inferior survival compared with all non-EZB patients.

## Predictive Genetic Biomarkers Using Serial Tumor Sampling in Patients with DLBCL

The afore discussed models predicting survival based on the molecular characterization of DLBCL utilize phenotypic and genetic traits available solely at diagnosis. However, recent technical progress enabled the detection of cell-free circulating tumor DNA (ctDNA) that is shed into the blood of lymphoma patients by tumor cells undergoing cell death [69]. This method captures the individual sequence variants of a DLBCL and tracks the specific genetic profile by targeted deep sequencing before, during and after therapy to quantitatively monitor treatment response, clonal evolution, and relapse of this aggressive lymphoma [70, 71, 72•, 73, 74••]. Studies using ctDNA profiling in DLBCL patients receiving immunochemotherapy demonstrated that the highly sensitive quantification of ctDNA level detected in blood of these patients was prognostic of the clinical outcomes [72•]. Notably, patients that showed a substantial decrease in ctDNA quantity after 1 cycle of therapy and/or after 2 cycles had superior outcomes compared to patients, for which a significant decline of ctDNA was not observed. These results prompted a novel survival prediction algorithm for DLBCL patients that integrated IPI, cell-of-origin classification, interim imaging as well as the analysis of pretreatment ctDNA levels and ctDNA decrease after the first and second cycle of therapy [74••]. Importantly, the combined survival prediction significantly improved on the individual risk predictors such as the established risk-factors (i.e., IPI, imaging, cell-of-origin classification). Future analyses combining the risk stratification including ctDNA analysis and genetic subclassification of DLBCL will likely elucidate further biological factors that dictate phenotype and clinical outcome of this aggressive disease.

## Conclusions

Over the last two decades, technical advances in genomic analyses allowed deep insights into the biology of DLBCL. In particular, tremendous progress was achieved decoding the vast heterogeneity of this aggressive lymphoma yielding in molecular subclasses that are biologically and clinically meaningful. The profound understanding of the molecular determinants that govern these varying phenotypes will enable the development of precision therapies. While many of these therapeutic strategies will be implemented in the future, the molecular subclassification allows prognostication of the clinical outcome already in current therapeutic settings. With genomics techniques becoming more widely available in clinical routine, the immediate task of next years will be to apply and test the applicable molecular prognostication methods in a real-world setting.
